# Impact of Orexin-A Treatment on Food Intake, Energy Metabolism and Body Weight in Mice

**DOI:** 10.1371/journal.pone.0169908

**Published:** 2017-01-13

**Authors:** Anne Blais, Gaëtan Drouin, Catherine Chaumontet, Thierry Voisin, Anne Couvelard, Patrick Christian Even, Alain Couvineau

**Affiliations:** 1 UMR Physiologie de la Nutrition et du Comportement Alimentaire, AgroParisTech, INRA, Université Paris-Saclay, Paris, France; 2 INSERM U1149/ Inflammation Research Center (CRI), Paris-Diderot University, DHU UNITY, Faculté de Médecine Site Bichat, 16, rue H. Huchard, Paris, France; Medical University of Vienna, AUSTRIA

## Abstract

Orexin-A and -B are hypothalamic neuropeptides of 33 and 28-amino acids, which regulate many homeostatic systems including sleep/wakefulness states, energy balance, energy homeostasis, reward seeking and drug addiction. Orexin-A treatment was also shown to reduce tumor development in xenografted nude mice and is thus a potential treatment for carcinogenesis. The aim of this work was to explore in healthy mice the consequences on energy expenditure components of an orexin-A treatment at a dose previously shown to be efficient to reduce tumor development. Physiological approaches were used to evaluate the effect of orexin-A on food intake pattern, energy metabolism body weight and body adiposity. Modulation of the expression of brain neuropeptides and receptors including NPY, POMC, AgRP, cocaine- and amphetamine related transcript (CART), corticotropin-releasing hormone (CRH) and prepro-orexin (HCRT), and Y_2_ and Y_5_ neuropeptide Y, MC_4_ (melanocortin), OX_1_ and OX_2_ orexin receptors (Y_2_R, Y_5_R, MC_4_R, OX_1_R and OX_2_R, respectively) was also explored. Our results show that orexin-A treatment does not significantly affect the components of energy expenditure, and glucose metabolism but reduces intraperitoneal fat deposit, adiposity and the expression of several brain neuropeptide receptors suggesting that peripheral orexin-A was able to reach the central nervous system. These findings establish that orexin-A treatment which is known for its activity as an inducer of tumor cell death, do have minor parallel consequence on energy homeostasis control.

## Introduction

Orexin-A and -B (also known as hypocretins 1 and 2) are hypothalamic neuropeptides of 33 and 28-amino acids, respectively, which are produced from the common 131- residue precursor, prepro-orexin [1]. Orexins are synthesized in the central nervous system mostly by the neurons of the lateral hypothalamus [2]. Both isoforms have been shown to be involved in multiple physiological processes including regulation of sleep/wakefulness states, energy balance, energy homeostasis, reward seeking and drug addiction [3, 4, 5]. Orexin-A regulates appetite, energy expenditure and metabolism [6]. Intra-cerebroventricular (i.c.v.) injections of orexin-A was shown to increase food intake in rats, while orexin-B was less effective [7]. Orexin system has a crucial role on sleep/wakefulness since orexin deficiency leads to narcolepsy and cataplexy in human and animals [8]. Recently, the U.S. Food & Drug Administration (FDA) approved the use of a reversible dual orexin receptor antagonist, Suvorexant, for insomnia [9]. Orexins and their functions have been mainly described in the central nervous system but orexins and their receptors are also detected in various organs including the intestine, pancreas, adrenal glands, kidney, adipose tissue and reproductive tract. However, their roles remain unclear [10, 11]. In peripheral tissues, orexins could affect insulin release, intestinal mobility, hormone secretion and blood pressure regulation [10]. Orexins trigger their central and peripheral effects by interacting with 2 members of the class A G-protein coupled receptors (GPCRs) family, i.e., orexin receptor-1 (OX_1_R) and orexin receptor-2 (OX_2_R) [1]. Basically, activation of these receptors by orexins induces intracellular calcium transients through G_q_-dependent and -independent pathways [12, 13, 3, 14, 15]. OX_1_R is more sensitive to orexin-A, whereas, the OX_2_R binds both orexin-A and orexin-B with the same affinity [16]. Moreover orexin-A, but not orexin-B is able to enter brain from blood by simple diffusion [17]. They provide strong output to the arcuate nucleus of the hypothalamus where they stimulate orexigenic neuropeptide Y/Agouti-related peptide (NPY/AgRP) [18] and the anorexigenic pro-opiomelanocortin (POMC) [19].

Apart from their effects on various physiological parameters, orexin-A and orexin-B can induce massive apoptosis in various colonic cancer cell lines, including HT-29, LoVo, Caco-2 and others cultivated in standard condition [20]. Moreover, in vivo, orexin-A injections can induce a strong inhibition of tumor growth in nude mice xenografted with these cell lines [20]. Orexin-A has also been reported to enhance SGC-7901 gastric cancer cells proliferation. However, the physiological significance of this result can be questioned because these cells were grown in serum free condition [21].

Taken together these results suggest that orexins may be helpful in cancer treatment. However, as orexins also stimulate feeding and orexin neurons respond to signals of metabolic status [22], the determination of the impact of chronic treatment by exogenous orexin-A peptide represent an important topic before considering the possibility of chronic orexin treatments. Therefore, the aim of the present work was to explore in healthy mice the consequences of orexin-A treatment at a dose similar to the one shown to be efficient to reduce tumor development in xenografted nude mice [20]. Physiological approaches were used to evaluate the effect of orexin-A on food intake pattern, energy metabolism body weight and body adiposity. Modulation of the expression of brain neuropeptides and receptors including NPY, POMC, AgRP, cocaine- and amphetamine related transcript (CART), corticotropin-releasing hormone (CRH) and prepro-orexin (HCRT), and Y_2_ and Y_5_ neuropeptide Y, MC_4_ (melanocortin), and OX_1_ and OX_2_ receptors (Y_2_R, Y_5_R, MC_4_R, OX_1_R and OX_2_R, respectively) was also explored. Our results, show that orexin-A treatment does not affect the energy expenditure components, and glucose metabolism but reduces intraperitoneal fat deposit and the expression of several brain neuropeptide receptors. These findings suggest that chronic treatment with orexin-A is sensed by the hypothalamus but has minor consequences on the control of energy homeostasis.

## Materials and Methods

### Animals

Sixteen, 8-week-old female Balb/C mice (Envigo, France) were housed at 22±1°C under a 12/12h reversed light/dark cycle (Lights on at 20:00) at four animals per cage. The mice were fed a standard AIN-93M diet containing by energy 20% of total energy as soy protein, 10% as fat and 70% as carbohydrate. The design of this study, conformed to the European legislation, was approved by the Animal Ethics Committee of INRA Jouy-en-Josas (Authorization number 13/012).

At ten weeks old, the mice were divided into 2 groups of eight mice. The control group received phosphate buffered saline (CT) and the second group received orexin-A (OxA) for 6 weeks. For treatment with orexin-A (GL Biochem, Shanghai, China), the peptide was diluted in phosphate buffer saline and 1 μmol/kg of body weight was administrated daily (at 09:00) by intraperitoneal (i.p.) injection in accordance with the result of a previous experiment in which it was shown that orexin-A was efficient to reduce tumor growth at a concentration as low as 0.1 μmol/kg and reached the greatest effect at 1 μmol/kg [20]. After 6 weeks, the mice were anesthetized with isoflurane, blood was drawn by cardiac puncture and the mice were immediately decapitated to ensure death. To avoid RNA degradation, the hypothalamus was immediately extracted from the fresh brain by making an incision medial to the piriform lobes caudal to the optic chiasma and anterior to the cerebral crus to a depth of 2–3 mm and was put directly in TRIzol reagent (Invitrogen, Breda, Netherlands), frozen in liquid nitrogen and stored at -80°C. Body composition was determined by dissection: liver, uterus, spleen, kidneys and pancreas, four white adipose tissue (WAT) pads (periovarian, retroperitoneal, mesenteric and total subcutaneous), interscapular brown adipose tissue (BAT) and the carcass (muscle and bone) were removed and weighed.

### Calorimetry and feeding patterns

The goal was to obtain for each mouse measures of food intake (FI) pattern, spontaneous physical activity (SPA), total and resting energy expenditure (TEE and REE) and respiratory quotient (RQ). Groups of 4 mice were housed at 18:00 in individual metabolic cages standing on an activity platform, bedded with wood litter and equipped with a weighed food cup. For gas analysis, the cages were multiplexed–all connected to the same gas analyzers. Thus oxygen consumption (VO_2)_ and carbon dioxide production (VCO_2_) were measured on each cage during 2min every 10min (2min for each cage, plus 2min on room air to correct values for room %O_2_ and %CO_2_). To reduce expenditure for thermoregulation (non-shivering thermogenesis), temperature in the room was maintained at 26–27°C in order to maintain in the metabolic cage a temperature of 27–28°C close to mouse thermoneutrality. Mice were housed during 3 days. Day 1 in the metabolic device was used for habituation. VO_2_–VCO_2_, FI and SPA were measured during day 2 and 3 and mean values were used for data analysis. For each cage FI and SPA were measured in 5s time bins. For analysis, data were pooled into 10min bins and combined with theVO_2_–VCO_2_ data. TEE was computed from VO_2_ and VCO_2_ according to the Weir formula [23, 24]. According to the fact that TEE = REE + SPA*(Cost of activity (Cost)), REE and Cost were computed by regression analysis between TEE and SPA, REE being the origin and Cost the slope of the regression (for details see [25]).

### mRNA extraction in the hypothalamus and Q-PCR experiments

The hypothalamus keep at -80°C were unfrozen just before mRNA extraction. Total RNA were extracted using TRIzol reagent, after homogenization using a Tissuelyser (Qiagen, Courtaboeuf, France), RNA concentrations in samples were measured on a NanoDrop ND-1000 UV-Vis spectrophotometer. RNA integrity was checked by ethidium bromide staining. 0.4 μg of total RNA in a final volume of 10 μl was reverse transcribed using a high-capacity cDNA archive kit protocol (Life technology, Courtaboeuf, France). Real-time PCR was performed to measure RNA expression using an ABI 7300 (Biosystems Life technology, Courtaboeuf, France) using Power SYBR Green PCR Master Mix, as previously described [26]. Reactions were performed as follows: denaturation for 10min at 95°C, 40 cycles at 95°C for 15s, followed by 1min at 60°C (amplification). Negative controls (reactions without reverse transcriptase or RNA) were used to monitor for contamination. The efficiency was estimated using a series of five-fold dilutions of the sample and checked for each run. A melting curve was performed to check for the absence of contamination. The primer sequences of target genes are given Table 1. Gene expression was calculated as 2^−ΔCT^. 18S and RPL13A were used as the housekeeping gene.

**Table 1:** Primer sequence use for brain mRNA Analysis.

Genes		sequence
POMC	Forward 5’	CTCCTGCTTCAGACCTCCATAGAT
	Reverse 5’	GGATGCAAGCCAGCAGGTT
CART	Forward 5’	CCGAGCCCTGGACATCTACTC
	Reverse 5’	AAATACTGACCAGCTCCTTCTCATG
AgRP	Forward 5’	GTTCCCAGAGTTCCCAGGTCTAA
	Reverse 5’	TTCTGCTCGGTCTGCAGTTG
NPY	Forward 5’	CTCTGCGACACTACATCAATCTCA
	Reverse 5’	GTGTCTCAGGGCTGGATCTCTT
CRH	Forward 5’	CAACCTCAGCCGGTTCTGA
	Reverse 5’	CCCCAGGCGGAGGAAGTA
MC_4_R	Forward 5’	TAGCCTGGCTGTGGCAGAT
	Reverse 5’	CGATGGTTTCCGACCCATT
Y_2_R	Forward 5’	CCGCTCCTGCTTCTGATCTC
	Reverse 5’	ACCCAAAGCAGGTCCGATT
Y_5_R	Forward 5’	AACCTTTGGCTCAGCATTGC
	Reverse 5’	CAGAGGGCCATGACTCAACA
OX_1_R	Forward 5’	CCGTCTACGCCTGCTTCAC
	Reverse 5’	AGGGTTGGCGGCACTGT
OX_2_R	Forward 5’	TGCAAAGACCAGAAGTACAACCA
	Reverse 5’	CAGATCCGAGCACGAAGGAA
HRCT	Forward 5’	TGGACCACTGCACTGAAGAGA
	Reverse 5’	CAGGGAACCTTTGTAGAAGGAAAG
18S	Forward 5’	GGGAGCCTGAGAAACGGC
	Reverse 5’	GGGTCGGGAGTGGGTAATTT
RPL13A	Forward 5’	GGATCCCTCCACCCTATGACA
	Reverse 5’	CTGGTACTTCCACCCGACCTC

### Oral glucose tolerance test

Oral glucose tolerance tests (OGTT) were performed after 6 weeks of treatment on 6 hours fasted mice. Andrikopoulos et al. previously shown that an OGTT test using a 2 g/kg glucose administered orally following 6 h of fasting is the best protocol to assess glucose tolerance in mice. [27]. At 8:00 food was removed and the orexin injections were done. At 14:00 the OGTT was performed. A bolus of glucose (2g/kg) was delivered in the stomach by a gavage needle. Blood samples were taken from the tail. 25 μl of blood was collected before gavage (t0), then 15, 30, 60 and 120 min after gavage for glucose and insulin determination. Blood glucose was measured immediately using an One Touch Vita (LifeScan, Issy-les-Moulineaux, France). The remaining blood was centrifuged (3000g, 15 min, 4°C) and plasma stored at -80°C until assayed for insulin using a Mercodia Mouse Insulin Elisa kit (Mercodia AB, Sweden).

### Immunohistochemical procedures

Pancreatic tissues were fixed overnight in 10% neutral buffered formalin, paraffin-embedded, and sectioned at 5 μm. The sections were stained with hematoxylin and eosin. For immunohistochemistry, after dewaxing, rehydrating tissue paraffin sections, and antigen retrieval by pretreatment with high temperature at pH 9, the sections were immunolabelled using an automated immunohistochemical stainer according to the manufacturer’s guidelines (Bond-Max slide stainer, Menarini, Leica Microsystems). For OX_1_R assessment, 3μm pancreatic tissue sections were incubated for 30 minutes with a polyclonal anti-OX_1_R antibody (Life technologies, Saint Aubin, France; polyclonal rabbit 1/100, PAS-33837), rinsed, and then incubated with a biotinylated secondary donkey anti-rabbit antibody diluted at 1:200. Sections were rinsed and incubated with Streptavidin (TrekAvidin-HRP; Biocare Medical) and diaminobenzidine ultraview detection kit (Bond Polymer Refine detection; DS9800; Leica Microsystems). Substitution of the primary antibody with PBS was used as a negative control.

### Statistics

Data are expressed as means ± SEM. Data were analyzed using the GLM procedure of SAS (version 9.1 SAS, Cary, NC). Post-hoc Tukey tests for comparisons between the two groups were performed. Significance was set at *P*<0.05. The *P* value is indicated when the difference was or tended (P<0.1) to be significant.

## Results

### Effect of orexin-A on food intake, body weight gain and, body composition

**Fig 1:**
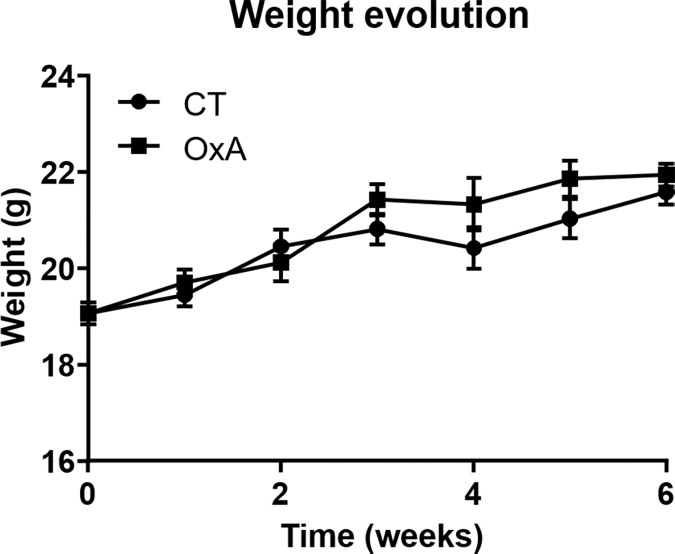
Effect of orexin-A injection on body weight evolution. Data are shown as mean ± SEM, n = 8. A two-factor repeated measures anova was performed to compare both groups and no significant difference was reported.

**Fig 2:**
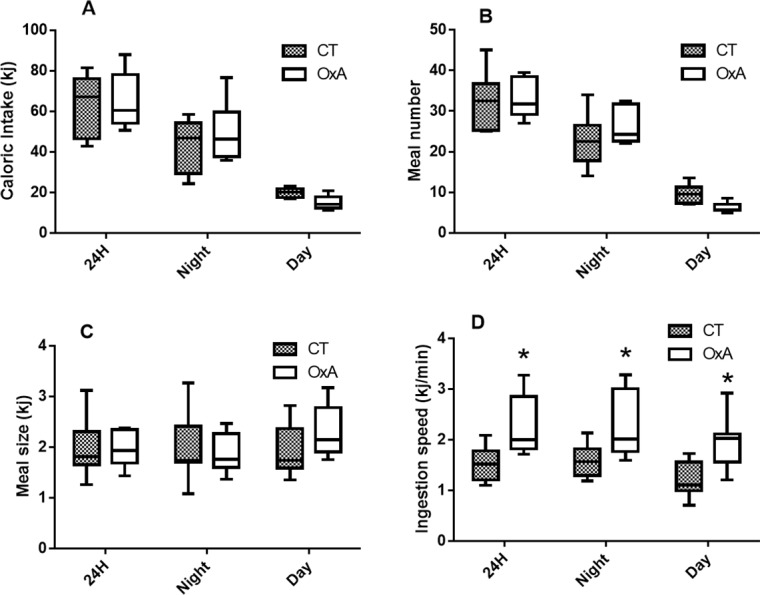
Components of caloric intake: total caloric intake (A), meal number (B) meal size (C) and Injestion speed (D). Data are presented as box and whiskers, n = 8. Significant differences are indicated: * *P*<0.05 (t-test).

Orexin-A injection did not affect body weight gain (Fig 1). This absence of effect was explained by the similar total caloric intake in control and orexin-A injected mice (Fig 2A). Moreover, meal number (Fig 2B) and meal size (Fig 2C) were similar in both groups. The only parameter significantly modulated by orexin-A treatment was the ingestion speed. Indeed, orexin-A mice ingested their food more quickly than control ones (Fig 2D).

**Fig 3:**
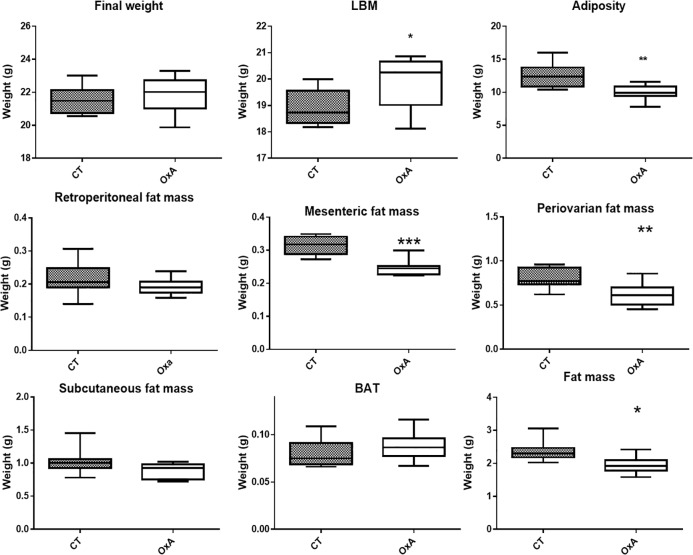
Effect of orexin-A injection on body composition evaluated immediatly after sacrifice. Data are presented as box and whiskers, n = 8. Significant differences are indicated *P<0.05, ** P<0.01, *** P<0.001 (Student-t test).

Orexin-A injection did not affect body weight, but increase lean body mass (LBM) and reduce the fat mass and the adiposity (Fat/LBM) (Fig 3). Detailed analysis of the fat mass (Fig 3) showed a decrease in periovarian and mesenteric fat masses while subcutaneous fat mass was not affected. The main element of body composition are presented in Table 2. Orexin-A treatment did not modulated organ weight however we observe a small but not significant increase of the carcass weight (CT 6.98 ± 0.29 vs. OxA 7.35 ± 0.44 g, P = 0.052) (Table 2) (S1 Fig). These results suggest that adipose tissue was sensitive to i.p. orexin-A administration in a site-specific manner. Consequently, orexin-A injection significantly reduced adiposity (fat/LBM).

**Table 2:** Body composition of the control and orexin-A-treated mice (values are in g).

	CT (Mean±SEM)	OxA(Mean±SEM)
Initial weight	19.04 ± 0.24	19.06 ± 0.26
Final weight	21.6 ± 0.76	21.93 ± 0.68
Delta weight	2.54 ± 0.20	2.58 ± 0.39
Carcass	6.98 ± 0.29	7.35 ± 0.44
Skin	2.13 ± 0.17	2.40 ± 0.19
Uterus	0.084 ± 0.011	0.092 ± 0.013
Liver	0.914 ± 0.045	0.901 ± 0.087
Spleen	0.103 ± 0.010	0.120 ± 0.022
Intestine	1.187 ± 0.121	1.108 ± 0.113
kidney	0.243 ± 0.013	0.252 ± 0.011

Values are means ± SEM, n = 8.

### Effect of orexin-A on the components of energy expenditure

**Fig 4:**
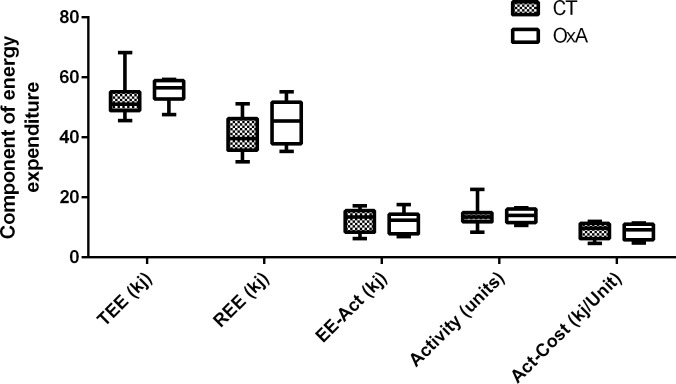
Components of energy expenditure: energy expenditure. TEE: total energy expenditure, REE: resting energy expenditure, EE-Act: energy expenditure spent in response to spontaneous motor activity, Activity: spontaneous motor activity, Act-Cost: energy cost of spontaneous activity. Data are presented as box and whiskers, n = 8. A t-test was performed and no significant differences are reported.

Orexin-A treatment did not affect total energy expenditure (TEE), resting energy expenditure (REE), spontaneous motor activity (Activity), energy cost of spontaneous activity (Act-Cost) and energy expenditure spent in response to activity (EE-Act) (Fig 4). Therefore, since food intake was also not significantly different, the decreased fat deposition in OxA mice resulted from changes in energy balance that were too small to be revealed by these measurements. Accordingly, the 0.24 g deficit in fat mass (4.67Kj) in orexin-A-treated mice along the 6 weeks of the study represent a daily deficit of 0.111 kJ, ie ~ 0.2% of the ~55 kJ daily energy expenditure, which is far below the sensitivity of food intake and calorimetry measurements (~5%). Taken together, these results indicate that chronic orexin-A treatment induced very tiny changes in daily energy balance resulting in a slight decrease in body fat over the long-term.

### Effects of orexin-A on the glucose and insulin responses to OGTT

**Fig 5:**
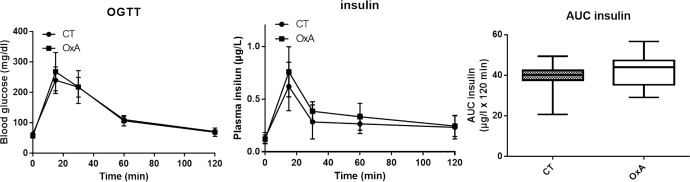
Blood glucose (A) and insulin (B) concentrations during the oral glucose tolerance test in control or orexin-A injected mice. Data are shown as mean ± SD, n = 8. A t-test was performed to compare both groups and no significant difference was reported. Insulin AUC were calculated using the trapezoidal rule (C). Data are presented as box and whiskers, n = 8. A t-test was performed and no significant differences are reported.

As acute orexin injection has been shown to decrease blood glucose [28], an oral glucose tolerance test (OGTT) was performed. Our results show that long term i.p. orexin-A injections at 1μmol/kg of weight did not significantly modify blood glucose response nor insulin secretion (Fig 5), indicating that chronic treatment with orexin-A did not affect insulin sensitivity and blood glucose regulation.

### OX_1_R expression in pancreas

**Fig 6:**
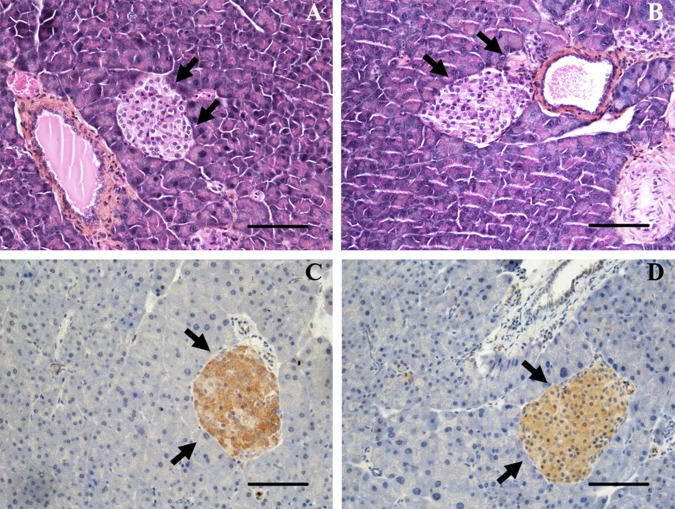
Histology of pancreas. Panel A and B, representative Hematoxylin and Eosin staining (H&E) of pancreas from control (A) and orexin-A-treated (B) mice. Panel C and D, representative OX1R immunostaining of pancreas from control (C) and Orexin-A-treated (D) mice. Arrows highlight the islets. Figures were taken at the same magnification (20x). Scale bar = 50 μm.

Previous report revealed the presence of OX_1_R immunoreactivity in pancreatic islets [19]. We investigated the effect of orexin-A chronic treatment on the histological aspect of pancreas. As shown in Fig 6A and Fig 6B, no differences were observed between control and orexin-A-treated pancreas. Indeed, we observed a normal aspect of the different cell types including acinar, duct, islet and endothelial cells in pancreas of both control and orexin-A-treated mice (black arrows, Fig 6A and Fig 6B). Furthermore, the islet number per pancreas (19 ± 2 islets and 21 ± 3 islets) was not significantly different (*P<0*.*617*, n = 8) in control and orexin-A treated mice. Similarly, the size of islets per pancreas determined as the percentage of islet surface versus the total surface of pancreas (0.60 ± 0.13% and 0.71 ± 0.11%) was not significantly different (*P<0*.*529*, n = 8) in control and orexin-A treated mice. Immunohistochemical staining indicated that OX_1_R was restricted to islets and not detected in acinar, duct or endothelial cells in control mice (Fig 6C) and treated mice (Fig 6D). Fig 6C and Fig 6D also show that chronic treatment by orexin-A had no impact on the immuno-detection of OX_1_R.

### Hypothalamus mRNA expression of neuropeptides and receptors involved in food intake

As circulating orexin-A has been shown to reach the brain we evaluated the expression of few neuropeptides and receptor involved in food intake control in the hypothalamus, one of the critical area of the brain that regulates this behavior. Orexin-A injection for 6 weeks did not affect the expression of mRNA encoding for preproorexin mRNA (HPRT), OX1R and Y5R and for NPY/AgRP and POMC/CART that are primarily involved in the control of food intake. However, CRH, Y_2_R and OX_2_R expressions were significantly decreased by i.p. orexin-A injections (Table 3) (S2 Fig). Similar results were obtained with RPL13A as housekeeping gene data are provided in a table provided as supplemental electronic attachments.

**Table 3:** Hypothalamus mRNA expression of neuropeptides involved in food intake.

	CT (Mean±SEM)	OxA(Mean±SEM)
POMC	6.78 ±2.28	4.63 ± 1.21
CART	1.40 ± 0.30	1.31 ± 0.22
AgRP	6.89 ± 1.05	5.95 ± 1.18
NPY	2.39 ± 0.62	1.91 ± 0.71
CRH	2.86 ± 0.54	1.83 ± 0.46**
MC_4_R	2.39 ± 0.30	2.15 ± 0.42
Y_2_R	6.10 ± 0.40	4.13 ± 1.00**
Y_5_R	10.50 ± 1.26	9.29 ±1.29
OX_1_R	4,68 ± 0.98	4.82±0.98
OX_2_R	3.55 ± 0.74	2.06±0.42**
HRCT	6.20 ± 0.39	5,57±1.10

mRNA expression (expression arbritrary units) of neuropeptides in the hypothalamus (expression relative to 18S). CRH, Y_2_R and OX_2_R were significantly reduce by orexin-A injections.Values are mean ± SEM, n = 8. A t-test was performed and significant differences are indicated *P<0.05,

** P<0.01.

## Discussion

Even if the half live of orexin-A is short 27 min [29], as the orexin-A concentration used was far larger than the physiological level measured in human blood (50 to 100 ng/ml) [30] or in mice brain (3 to 6 pmol/g wet weight) [16], it was thus important to explore the consequences of long-term orexin-A treatment on the components of energy balance in healthy mice. The present study strongly suggests that daily i.p. injections of orexin-A at 1 μmol/kg, the most efficient concentration to reduce tumor growth in mice [20], despite the fact that it modulated the expression of few brain neuropeptide receptors in the hypothalamus, did not induce major effects on the various components of energy expenditure and on long-term body weight evolution. However, we report that orexin-A-treated mice ingested their food more quickly than control ones this results is in accordance with a recent study showing that intranasal administration of orexin-A to rats was able to increase food intake during the first 4 hours just after the administration but as we shown, the total amount of food consumption over 24 hours was not increase [31]. A small but significant decrease in visceral fat masses was observed at the end of the 6 weeks treatment. These findings establish that orexin-A treatments at a dose that can induce death of tumor cells, do have minor consequence on the control of energy homeostasis.

Several studies have shown that G protein-coupled receptors (GPCRs) represent new promising targets for the therapeutic treatment of various cancers [32]. It has been shown in colon cancers which ectopically expressed OX_1_R, that orexins induced a robust mitochondrial apoptosis [33] This pro-apoptotic effect was also shown in other cancer cell lines derived from human neuroblastoma (SK-N-MC cell line) [33]. Voisin et al. [20] previously showed that OX_1_R are also expressed in all resected primary colorectal tumors and liver metastases tested, but OX_1_R were not present in normal colon tissues. These results suggested that the orexin-A/OX_1_R system might depict a new promising target in colon cancer therapy, and probably in other cancers including prostate cancer [34]. It appears therefore important to test the long-term effects of chronic orexin-A treatment on caloric intake, energy expenditure and energy balance to assess the safety of long-term treatments which are well known target of orexins.

Our results show that orexin-A injection increased ingestion speed but did not significantly affect daily food intake, meal size and meal frequency. Moreover, we report no abnormality of spontaneous physical activity and glucose homeostasis and no changes in any of the components of total energy expenditure. This absence of modulation is observed despite a slight modification of the expression of neuropeptides and receptors in the mice hypothalamus, one of the critical area of the brain involved in the regulation of energy balance. Thus, we observed in the hypothalamus a decrease of CHR, Y_2_R and OX_2_R (but not OX_1_R) mRNA expression, suggesting that, as previously shown [17], a small fraction of peripheral orexin-A was able to cross the blood-brain barrier. The lower CRH mRNA expression observed in orexin-A-treated mice also confirms the role of orexins in the control of CRH neurons [35].

After 6 weeks, the weight of orexin-A injected mice was not significantly different from the weight of control mice, which is in agreement with previous studies showing that chronic i.c.v. injections do not result in body weight gain [36]. However, we observed a small but significant reduction of visceral fat mass and adiposity but we did not observe any decrease of the subcutaneous fat, suggesting a lesser sensitivity of these fat pads to orexin-A. These results are supported by previous works who showed that in human adipocytes isolated from subcutaneous compared to intra-abdominal adipose tissue, orexin-A had different actions on expression of key genes involved in adipogenesis and on adipocyte metabolism which can explain why orexins are able to reduce adipogenesis in intra-abdominal but not in subcutaneous adipocytes [37]. However, we cannot definitely discard a possible local effect due to the fact that orexin-A was i.p. injected. Taken together these results support the potential antiobesity effects of orexins reported in animals overexpressing orexin or its receptors [38]. Moreover, studies using genetic models also showed that higher orexinergic signaling provides resistance to the development of obesity supporting the view that the orexin system can control the energy system and obesity [39, 40, 41, 42].

It should be noted that chronic treatment of mice with orexin-A seems to have no impact on the cellular structure of pancreas. We also did not report any modification of insulin secretion and glucose homeostasis. As OGTT was performed 6 hours after the daily i.p. injection, our study cannot exclude an acute effect of orexin-A injection on blood glucose as previously reported [28]. However, our results support that if orexin-A can have an acute effect, the long-term treatment has no significant consequence on blood glucose regulation and insulin sensitivity. Many *in vivo* studies tried to evaluate the effect of orexins on the pancreas but conflicting results have been reported depending on the conditions, and significant modulation of insulin or glucose plasma were not always observed [29, 43, 44, 45, 46]. Our results thus support previous *in vivo* data showing that orexin-A does not appear to be involved in the regulation of glucose metabolism.

In conclusion, the present study suggests that increased peripheral orexin-A induced by daily i.p. injections were sensed by the hypothalamus and affected the expression of several receptors and neurotransmitters but did not result in any important effects on energy intake and energy expenditure possibly as a result of its short (30 min) half-life in the plasma. Since this same treatment as been reported to have strong anti-tumoral properties, our data support further development for the use of this compound in human anti-cancer therapy. In this context, the development of orexin analogs with stronger stability would be a valuable challenge [47].

## Supporting Information

S1 Fig Effect of orexin-A on body composition. Data are presentredas box and whiskers, n = 8. A t-test was performed and significant differences are indicated *P<0.05, **P<0.01 (TIF).
(TIF)

S2 Fig Hypothalamus mRNA expression of neuropeptides involved in food intake. mRNA (expression arbitrary units) expression of neuropeptides in the hypothalamus(relative to 18S). CRH, Y_2_R and OX_2_R were significantly reduce by orexin-A injections. Data are presented as box and whiskers. A t-test was performed and significant differences are indicated *P<0.05, **P<0.01 (TIF).
(TIF)

S1 Table Effect of orexin-A on weight evolution, caloric intake, energy expentidure, glucose and insulin values, Ox1R expression in pancreas and hypothalamus gene expression of neuropeptides and receptors involved in food intake. n = 8. A t-test was performed and significant differences are indicated *P<0.05, **P<0.01 (data orexin A dec 6.xlxs).
(XLSX)
