# Supplementary figures and images for: Impact of Orexin-A Treatment on Food Intake, Energy Metabolism and Body Weight in Mice
Source: PLoS One. 2017 Jan 13;12(1):e0169908. doi: 10.1371/journal.pone.0169908 (PMC5235373; doi:10.1371/journal.pone.0169908)

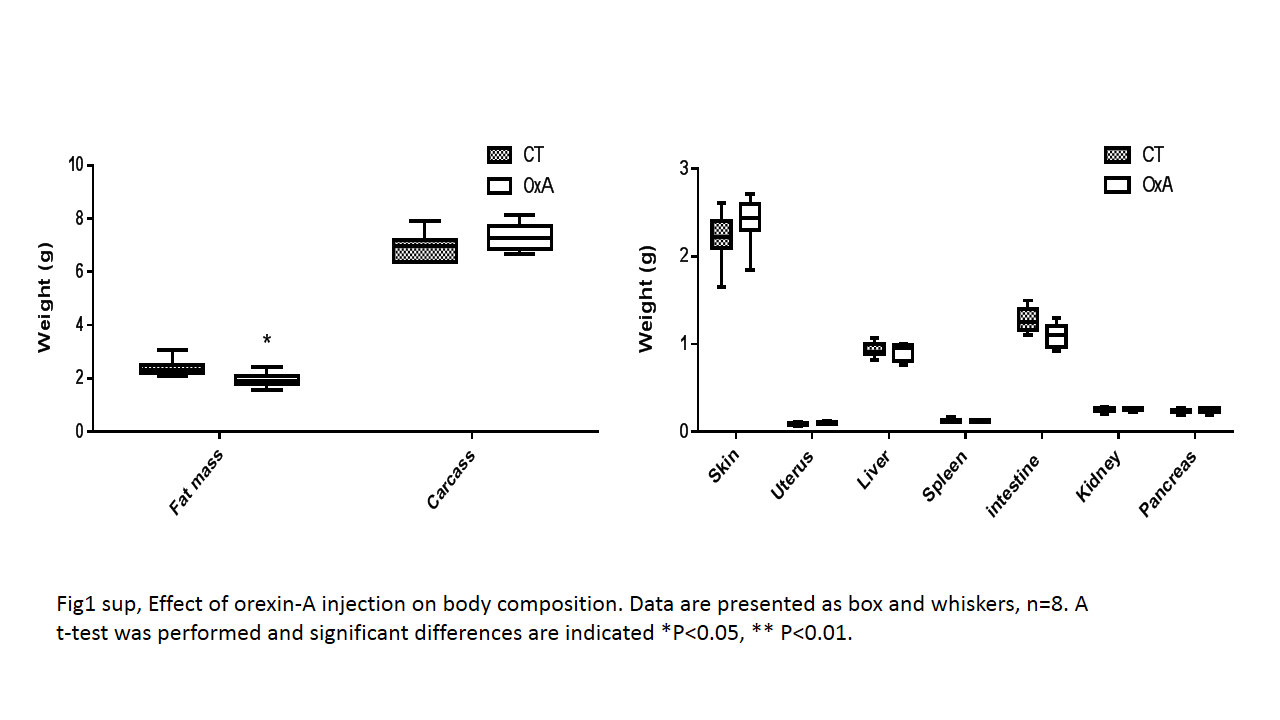

Supplement: S1 Fig — Data are presentredas box and whiskers, n = 8. A t-test was performed and significant differences are indicated *P<0.05, **P<0.01 (TIF). (TIF) [file pone.0169908.s001.tif]

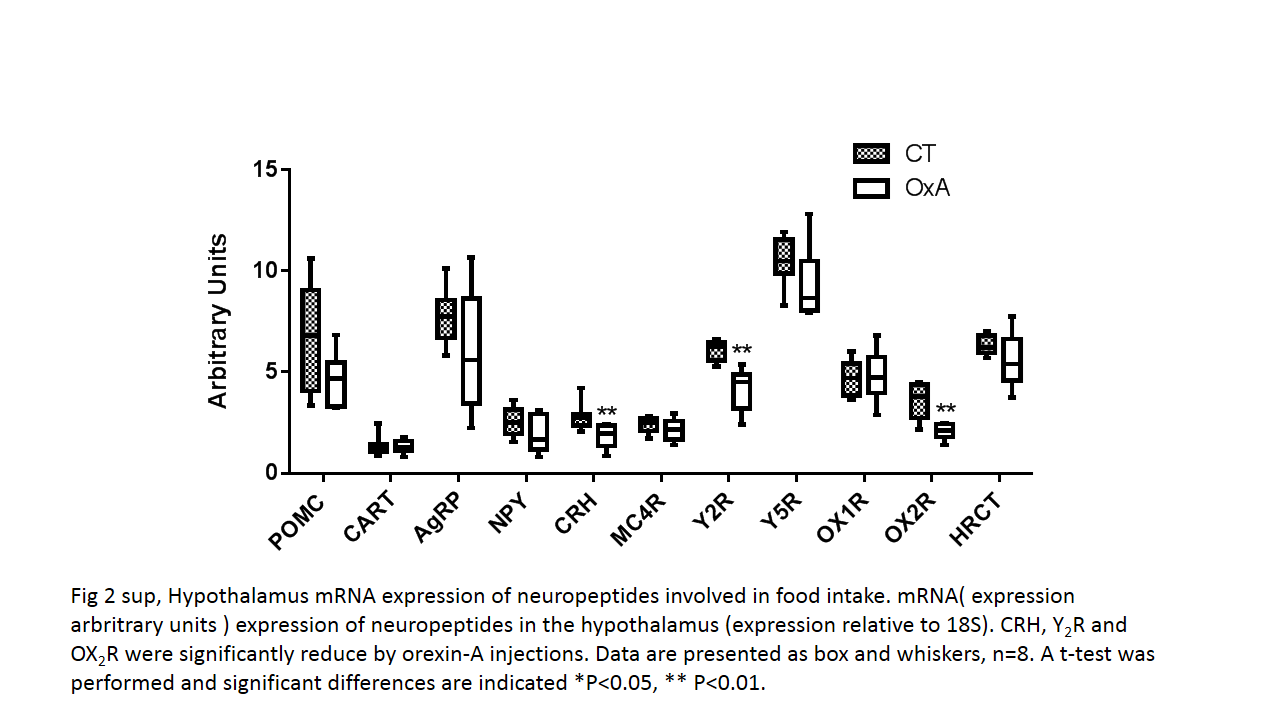

Supplement: S2 Fig — mRNA (expression arbitrary units) expression of neuropeptides in the hypothalamus(relative to 18S). CRH, Y2R and OX2R were significantly reduce by orexin-A injections. Data are presented as box and whiskers. A t-test was performed and significant differences are indicated *P<0.05, **P<0.01 (TIF). (TIF) [file pone.0169908.s002.tif]
